# Hemoperfusion leads to impairment in hemostasis and coagulation process in patients with acute pesticide intoxication

**DOI:** 10.1038/s41598-019-49738-1

**Published:** 2019-09-16

**Authors:** Samel Park, Md-Imtiazul Islam, Ji-Hun Jeong, Nam-Jun Cho, Ho-yeon Song, Eun-Young Lee, Hyo-Wook Gil

**Affiliations:** 1 Department of Internal Medicine, Soonchunhyang University Cheonan hospital, Cheonan, Korea; 2 Department of Microbiology and Immunology, Soonchunhyang University Medical College, Cheonan, Korea; 3 Soonchunhyang Institute of Medi-Bio Science, Soonchunhyang University, Cheonan, Korea

**Keywords:** Adverse effects, Renal replacement therapy

## Abstract

Hemoperfusion (HP) is one of the important treatment modalities in extracorporeal therapy for patients with acute intoxication. Its use has declined during the past 20 years despite its efficacy, because of its side effects, especially an increased risk of bleeding. Mechanisms of hemostasis impairment have not been clearly elucidated and studies demonstrating the mechanism are lacking. It is not clear which step of the hemostatic process is impaired during HP, and whether it leads to an increased risk of bleeding. We performed both *in vivo* and *in vitro* studies to elucidate the mechanism of impairment in the hemostatic process. In patients with acute pesticide intoxication who underwent HP, the platelet count decreased rapidly during the first 30 minutes from 242.4 ± 57.7 × 10^3^/μL to 184.8 ± 49.6 × 10^3^/μL, then gradually decreased even lower to 145.4 ± 61.2 × 10^3^/μL over time (p < 0.001). As markers of platelet activation, platelet distribution width increased continuously during HP from 41.98 ± 9.28% to 47.69 ± 11.18% (p < 0.05), however, mean platelet volume did not show significant change. In scanning electron microscopy, activated platelets adhered to modified charcoal were observed, and delayed closure time after HP in PFA-100 test suggested platelet dysfunction occurred during HP. To confirm these conflicting results, changes of glycoprotein expression on the platelet surface were evaluated when platelets were exposed to modified charcoal *in vitro*. Platelet expression of CD61, fibrinogen receptor, significantly decreased from 95.2 ± 0.9% to 73.9 ± 1.6%, while those expressing CD42b, von Willebrand factor receptor, did not show significant change. However, platelet expression of CD49b, collagen receptor, significantly increased from 24.6 ± 0.7% to 51.9 ± 2.3%. Thrombin-antithrombin complex, a marker for thrombin generation, appeared to decrease, however, it was not statistically significant. Fibrin degradation products and d-dimers, markers for fibrinolysis, increased significantly during HP. Taken together, our data suggests that hemoperfusion leads to impairment of platelet aggregation with incomplete platelet activation, which was associated with reduced thrombin generation, accompanied by increased fibrinolysis.

## Introduction

Hemoperfusion (HP) is one of the important modalities in extracorporeal treatment for patients with acute intoxication^1^. Although HP can remove both protein-bound and lipid-soluble materials^2–4^, the use has declined during the past 20 years, despite its efficacy^5^. One of the reasons for the declining use of HP is its side effects, especially increased risk of bleeding caused by decreased platelet count and impaired coagulation. Our institution previously reported that systemic bleeding was observed in 3% of patients with acute intoxication who underwent HP, and that it was an important factor associated with mortality^1^. The average platelet counts has also been reported to decrease about by 30% per HP session^6^.

Mechanisms of HP-induced thrombocytopenia and impairment of the hemostatic process have not been clearly elucidated. Efforts to evaluate causes of thrombocytopenia or bleeding tendency developing after HP began with a very small number of studies, until the 1980s, when HP was replaced with hemodialysis in patients with renal failure and progress on these studies stopped. However, the mechanisms can be inferred through previous studies on several hemostatic impairments caused by direct blood-contacting devices, such as extracorporeal membrane oxygenation, and ventricular assist devices. It is well-described in the literature that the use of equipment which directly contacts the blood increases not only the risk of bleeding, but paradoxically, also the rate of thrombosis^7–9^.

Although it is not clear which step is impaired in the hemostasis process during HP and whether it leads to an increased risk of bleeding, we assumed that the mechanisms of bleeding complications induced by HP are similar to those induced by direct blood-contacting devices. In order to clarify the pathophysiology, it is necessary to identify which step of the hemostatic process is affected by HP. The present study was conducted using representative markers of each step in the hemostatic process in order to determine which step of process was been impaired.

## Materials and Methods

Between January and December 2016, 250 patients with acute pesticide intoxication from ingestion of undiluted pesticides were treated at the Nephro-toxicology Division of Soonchunhyang University Cheonan Hospital (Cheonan, Korea). Patients were excluded from this study for anti-platelet drug use, active cancer, hematological or hemostatic disorders, treatment ≥12 hours after ingesting pesticide, transfer from another hospital after undergoing HP or HD, and those critically unstable where HP could not be performed. A clinician in charge of the emergency room made the decision whether or not to perform HP. In this study, 25 patients were included.

HP was performed as previously reported^6^. Briefly, an Adsorba 300 C HP cartridge (Baxter, KG Hechingen, Germany), which has modified charcoal adsorbent coated with cellulose, with a 300 m^2^ surface area was used. Heparin was infused with loading and maintaining doses of 2,000 IU and 1,000 IU/h, respectively. HP was conducted for three hours with 200 ml/min of blood flow.

White blood cells (WBC), platelet count, hemoglobin (Hb), platelet distribution width (PDW), mean platelet volume (MPV), and plateletcrit (PCT) were measured with an auto-analyzer (ADVIA-2120, Siemens Diagnostics, Tarrytown, New York, USA). The instrument was calibrated and samples were processed according to the manufacturer’s instructions. Previous studies on the effects of dialysis on platelets have reported that platelet counts typically fall during the first 15–30 min of dialysis^10^. Based on the previous studies on HD and the nature of HP, during which the highest initial removal rate occurs at the initiation of HP and continues to decrease over time due to a saturation of capacity, it was speculated that the acute decrease in platelets during the earlier phase of HP would be more prominent than in HD. Thus, blood was drawn from the inflow line of the HP apparatus at the initiation of HP, and 30, 60, and 180 minutes thereafter. To assess whether a hemostatic process became impaired after HP, a commercial platelet function assay, PFA-100 (Dade PFA Collagen/EPI and Collagen/ADP test cartridges, Siemens Healthcare, Marburg, Germany) was used. The results of PFA-100 were simply compared before and after HP.

To evaluate the effect of modified charcoal on platelet dysfunction, changes in the expression of platelet surface glycoproteins were evaluated during modified charcoal exposure *in vitro*. Three glycoprotein monoclonal antibodies (Platelet GP screening kit 13010, BioCytex, Marseille, France), representing each step of primary hemostasis, including platelet adhesion (CD49b, collagen receptor), activation (CD42b, von Willebrand factor [vWF] receptor), and aggregation (CD61, fibrinogen receptor) were used.

Human platelets for the *in vitro* study were prepared by the blood bank at our institution as a form of platelet-rich plasma (PRP). First, PRP was gently rinsed twice with normal saline to remove as much remaining plasma, especially vWF, other procoagulants, and citrate that inevitably administered to store platelet, as possible. Addition of normal saline to the rinsed PRP followed to dilute the platelet count to a normal human platelet count. Then, PRP was divided into 10 mL aliquots, added to 50 mL conical tubes with 20 g aliquots of modified charcoal obtained from an Adsorba 300C HP cartridge, and the tubes were mixed and gently shaken for 30 minutes to evaluate the effect of modified charcoal on the expression of platelet glycoproteins.

Platelet glycoprotein levels on the surface were then assayed with flow cytometry using the monoclonal antibodies to glycoprotein described above. The glycoproteins expressed on the surface of platelets were extracted and analyzed using a FACS Canto II flow cytometer (BD Biosciences, Franklin Lakes, NJ, USA). The samples for flow cytometry were immuno-labeled and stained with fluorescent dye at room temperature for 10 minutes each, according to the manufacturer’s instructions. Data were analyzed using FlowJo^®^ software (TreeStar Inc., Ashland, OR, USA). This *in vitro* study was performed in triplicate.

The conical tubes with 10 ml of PRP and 20 g of modified coated were prepared as described above, then mixed and shaken for 30, 60, or 120 minutes. Then, the surface of the modified charcoal was evaluated using scanning electron microscopy (SEM). The modified charcoal in a HP cartridge used to treat a patient with pesticide intoxication was also evaluated using SEM. Platelets on cellulose-coated charcoal were fixed in 2.5% glutaraldehyde in a phosphate buffer (0.1 mol, pH 7.4) for 4 hours and rinsed 3 times in phosphate buffer for 5 minutes, before fixation for 1 hour with 1% osmium tetroxide. The samples were then rinsed 3 times for 5 minutes with distilled water and dehydrated serially in 50%, 70%, and 90% ethanol, and twice in 100% ethanol. Samples were then dried with tetramethylsilane coated with platinum (Cressington 108 Auto, Cressington Scientific Instruments UK, Watford, UK), and observed via a SEM (JSM-6701F, JEOL, Tokyo, Japan) at an acceleration voltage of 2 kV. Images were obtained at multiple magnifications on each sample’s surface. In addition to images of cellulose-coated charcoal obtained from the *in vitro* study, images obtained after cutting a HP cartridge used in an actual patient with pesticide intoxication were also evaluated.

To study the effect of HP on the coagulation process, the levels of thrombin-antithrombin complex (TAT), fibrinogen degradation products (FDP), and d-dimers were measured, using a commercial enzyme-linked immunosorbent assay kit (Abcam, Cambridge, United Kingdom) and automated latex-enhanced immunoassay kits (HemosIL FDP kit and D-Dimer HS kit, Instrumentation Laboratory, Barcelona, Spain), respectively. In contrast to changes in platelet counts, we hypothesized that the influence on the coagulation process by HP occurred continuously, and would result in linear changes of TAT, FDP, and d-dimers. Therefore, blood samples were acquired at the initiation of HP, and at 60, 120, and 180 minutes thereafter, in other patients who were not involved in the *in vivo* study described above.

The study was conducted in accordance with the Declaration of Helsinki, and the protocol was approved by the Institutional Review Board of the Soonchunhyang University Cheonan Hospital (IRB no. 2013-12-010). All study participants provided informed written consent prior to study enrollment. Some participants were unable to consent to the study due to their level of consciousness. In those cases, written informed consent was obtained from the next of kin.

### Statistical analysis

The Friedman test and the Wilcoxon sign-rank test with Bonferroni correction were used to compare WBC, Hb, platelet counts, MPV, PDW, PCT, TAT, FDP, and d-dimer levels, as appropriate. The paired *t*-test was used to compare the results from flow cytometry. Data with normal distribution were expressed as mean ± standard deviations (SD), otherwise it was expressed as median (25 percentile – 75 percentile). In addition, data in the coagulation study of only seven cases were expressed as mean ± standard errors (SE).

All variables with a *p*-value < 0.05 in the univariate analysis were considered statistically significant. Data were analyzed using R software, version 3.4.3 (The R Foundation for Statistical Computing, Vienna, Austria), SPSS software, version 24.0 for Windows (SPSS, Inc., Chicago, IL, USA).

## Results

### Basal clinical and laboratory characteristics

Changes in WBC counts, Hb, platelet counts, PDW, MPV, and PCT during HP were assessed in 25 patients. Basal laboratory data from the emergency department are summarized in Table 1. In 14 cases, the type and volume of ingested pesticide were identified. In the other cases, however, information about the ingested pesticide was unavailable. In these patients, acute pesticide intoxication was suspected because of the smell from the patients when they were discovered by other people. Of the 25 patients, three patients eventually died. All patients received care following the treatment protocol of our institution.

**Table 1:** Basal clinical and laboratory characteristics (n = 25).

Characteristics	Values
Age, year	55.8 ± 16.9
Male sex, number (%)	11 (44)
Ingested volume, ml^†^	237.9 ± 139.7
**Type of pesticide**	
*Organophosphate*	2
*Other insecticide*	3
*Glyphosate*	4
*Glufosinate*	2
*Other herbicide*	2
*Fungicide*	1
*Unknown type*	11
WBC count, /μl	10957.6 ± 5391.1
Hemoglobin, g/dl	12.9 ± 1.9
Platelet count, ×10^3^/μl	242.4 ± 57.7
Protein, g/dl	7.1 ± 0.7
Albumin, g/dl	4.3 ± 0.5
Glucose, mg/dl	160.8 ± 66.0
Urea nitrogen, mg/dl	13.3 ± 4.1
Creatinine, mg/dl	0.80 (0.65–1.15)
Sodium, mEq/l	140.8 ± 2.3
Potassium, mEq/l	3.8 ± 0.6
Chloride, mEq/l	101.9 ± 3.9
Calcium, mg/dl	9.2 (8.5–9.5)
Phosphorus, mg/dl	3.9 ± 1.7
Uric acid, mg/dl	4.9 ± 1.4
Creatine kinase, IU/l	91 (63–170)
pH	7.3 ± 0.2
PaO_2_, mmHg	89.8 (78.5–104.0)
Base excess	−5.9 ± 6.2

Abbreviations: WBC, white blood cell; pH, potential hydrogen; PaO_2_, partial pressure of oxygen.

^†^The volume and type of ingested pesticide could be identified in only 14 cases.

### Changes in complete blood counts and platelet indices

**Figure 1:**
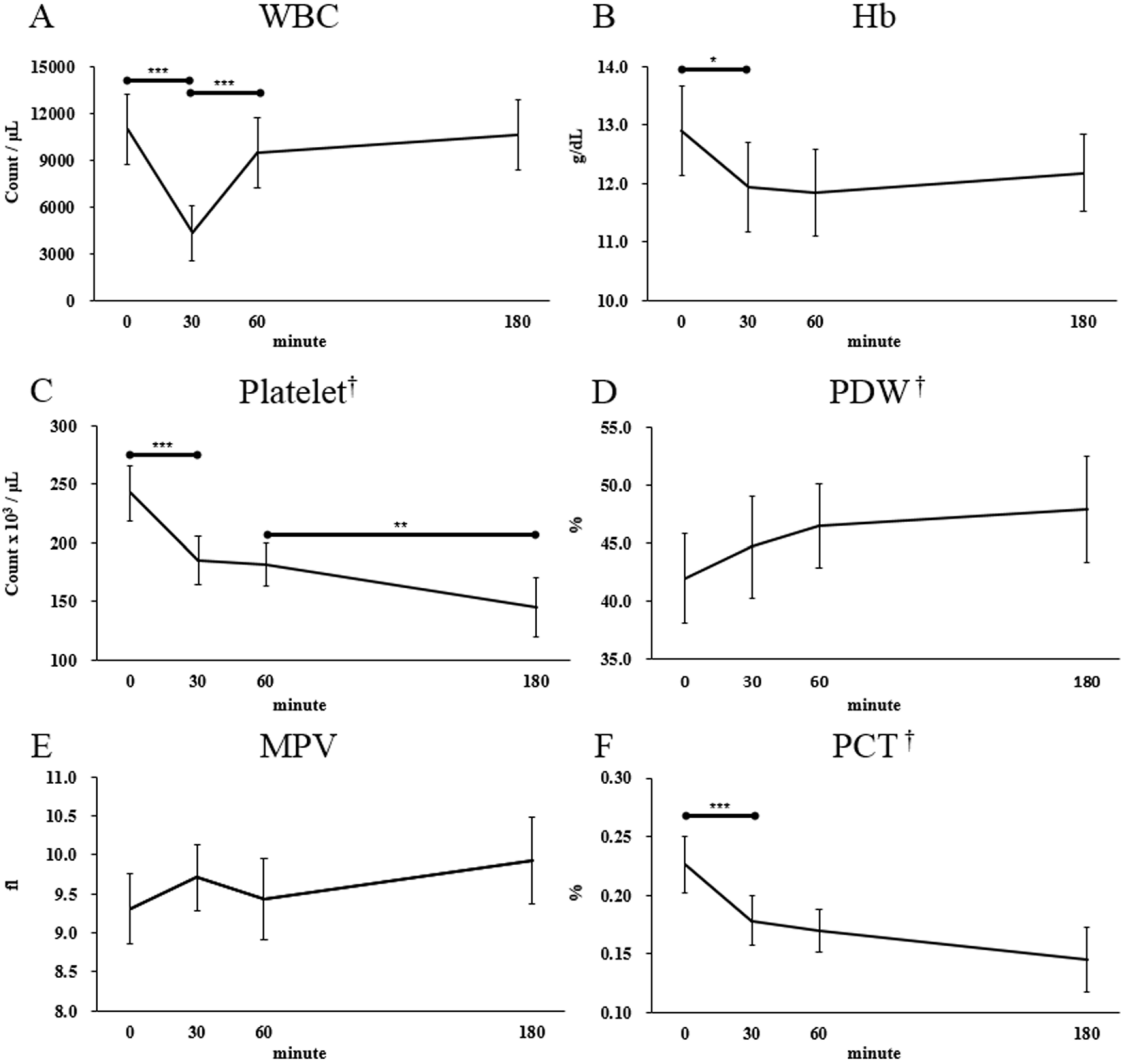
Changes in complete blood counts and platelet indices during hemoperfusion. (**A**) White blood cell count. (**B**) Hemoglobin. (**C**) Platelet count. (**D**) Platelet distribution width. (**E**) Mean platelet volume. (**F**) Plateletcrit. *p < 0.05; **p < 0.01, ***p < 0.001 in repeated measures ANOVA or Friedman test with Bonferroni correction. ^†^*p* < 0.05 in Jonckheere-Terpstra trend test.

WBC counts decreased rapidly during the first 30 minutes and then increased again for 30 minutes. Recovered WBC counts were sustained until the end of HP (Fig. 1A). Hb levels fell significantly during the first 30 minutes after initiation of HP and the decreased levels were sustained until the end of HP (Fig. 1B). The basal platelet counts and PCT levels were 242.4 ± 57.7 × 10^3^/μL and 0.230 (0.175–0.265) %, respectively, then rapidly decreased during the first 30 minutes to 184.8 ± 49.6 × 10^3^/μL and 0.170 (0.140–0.195) %, respectively. After that time, the counts decreased much lower to 145.4 ± 61.2 × 10^3^/μL and 0.140 (0.085–0.195), respectively (Fig. 1C,F). The trend test showed that they gradually declined over time during HP (platelet counts, *p* < 0.001; PCT, *p* < 0.001). As markers of platelet activation^11^, PDW gradually increased with time from 41.98 ± 9.28% to 47.69 ± 11.18% (Fig. 1D), however, MPV did not show significant changes (Fig. 1E).

### Changes in closure time measured using PFA-100

**Figure 2:**
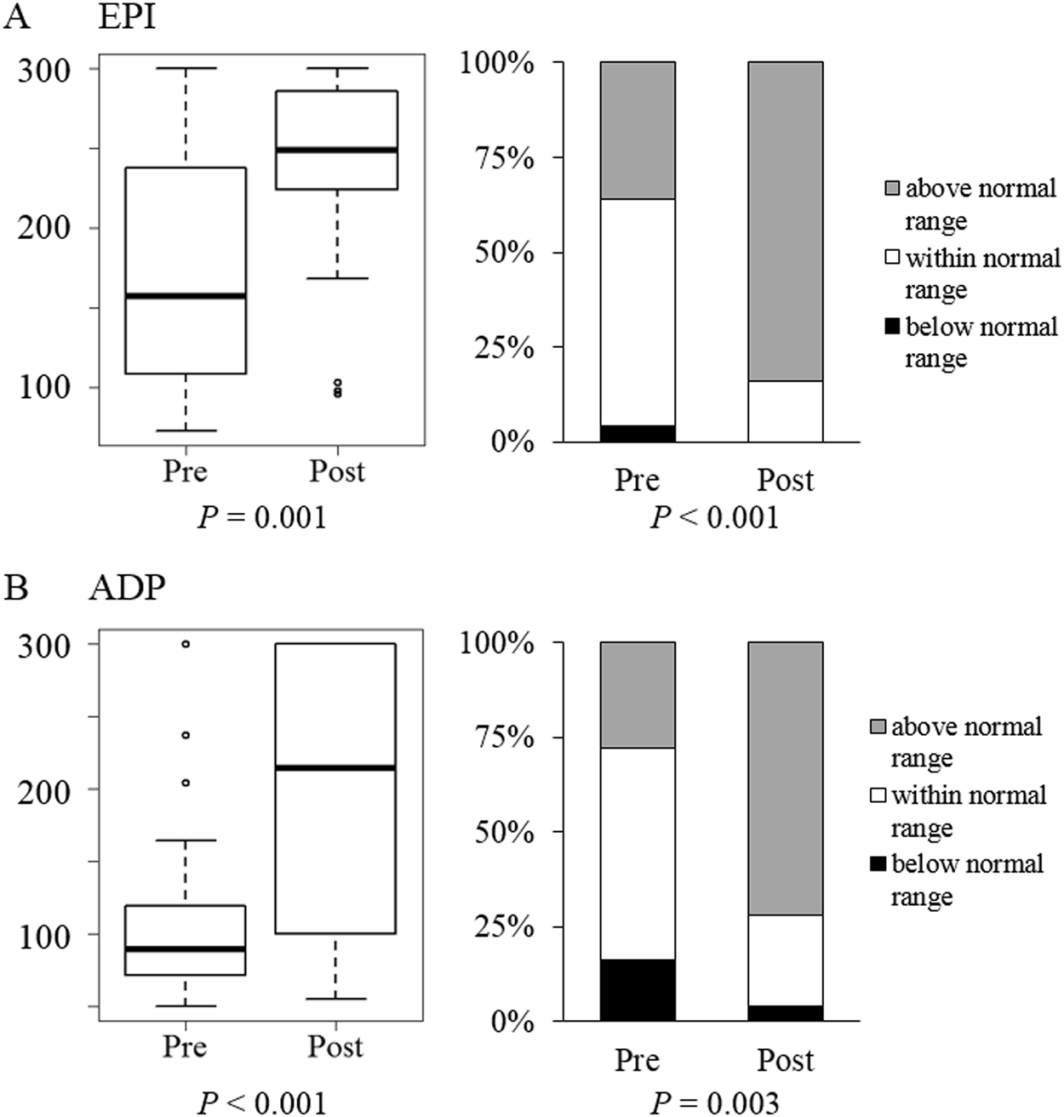
Changes in closure time measured using PFA-100 during hemoperfusion. (**A**) EPI coated cartridge. (**B**) ADP coated cartridge.

The PFA-100 test measure closure time in cartridges, of which membrane coated with epinephrine or ADP. Delayed closure time suggests platelet dysfunction^12^. Since PFA-100 does not measure closure times longer than 5 minutes^12^, statistical analysis was done in two ways, as categorical and continuous variables. Figure 2 shows changes of closure times before and after HP, with closure times using cartridges coated with epinephrine or ADP at each time. In both, the absolute closure times and the proportion of closure times above the normal range increased significantly. These results suggest that platelet function was impaired by exposure to the extracorporeal circuit during HP^13^.

### Scanning electron microscopy of the surface of modified charcoal

**Figure 3:**
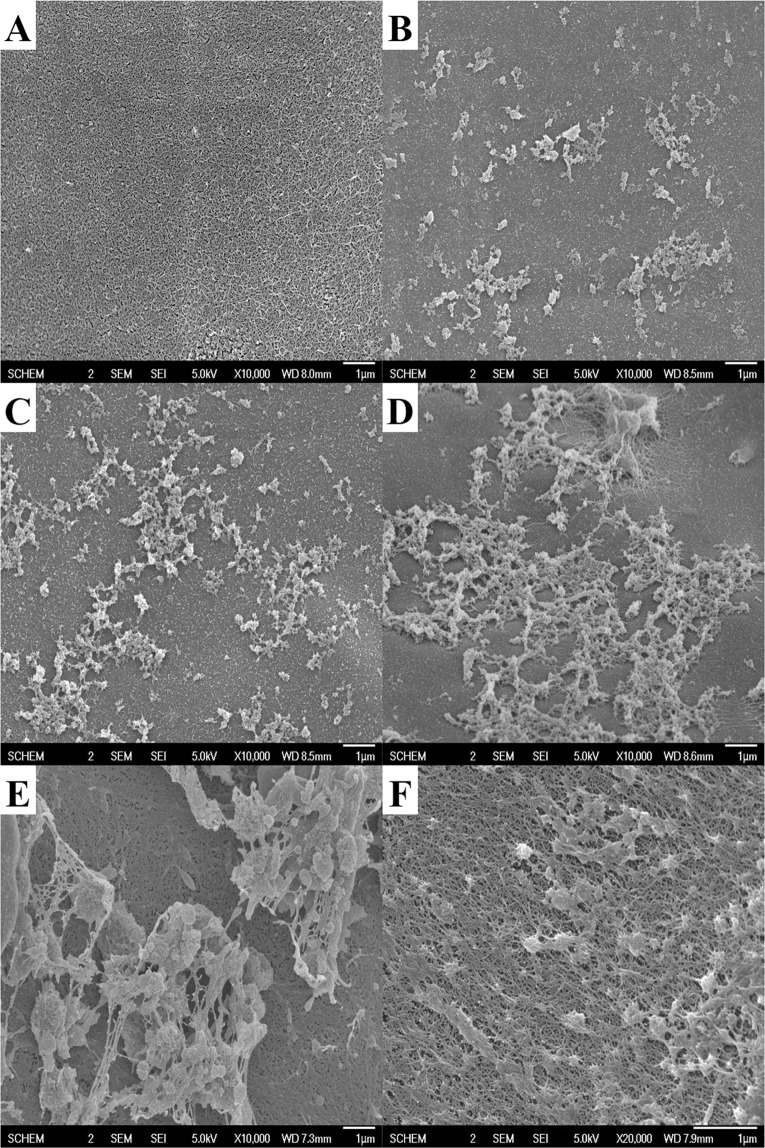
Scanning electron microscopy of the surface of modified charcoal from hemoperfusion cartridge. (**A**) Clear surface of the modified charcoal coated with cellulose, obtained from a non-used HP cartridge. Activated platelets adhered to the surface of modified charcoal after (**B**) 30 min, (**C**) 60 min, and (**D**) 120 min of shaking *in vitro*. (**E**,**F**) Surface of modified charcoal obtained from a HP cartridge used in a patient with pesticide intoxication. (**E**) Red blood cells and activated platelets adhered to the surface of modified charcoal. (**F**) The activated platelets and fibrin clots adhered to the surface of modified charcoal.

Results of SEM evaluation of the modified charcoal obtained from the *in vitro* experiment showed that platelets aggregated on the surface of modified charcoal gradually increased over time (Fig. 3B–D). When the modified charcoal obtained from HP used in an actual patient was evaluated using SEM, aggregated red blood cells (Fig. 3E) and platelets aggregated with fibrin on the modified charcoal surface were observed (Fig. 3F). These results suggest that platelet activation occurred during HP.

### Changes in the expression of platelet surface glycoprotein

**Figure 4:**
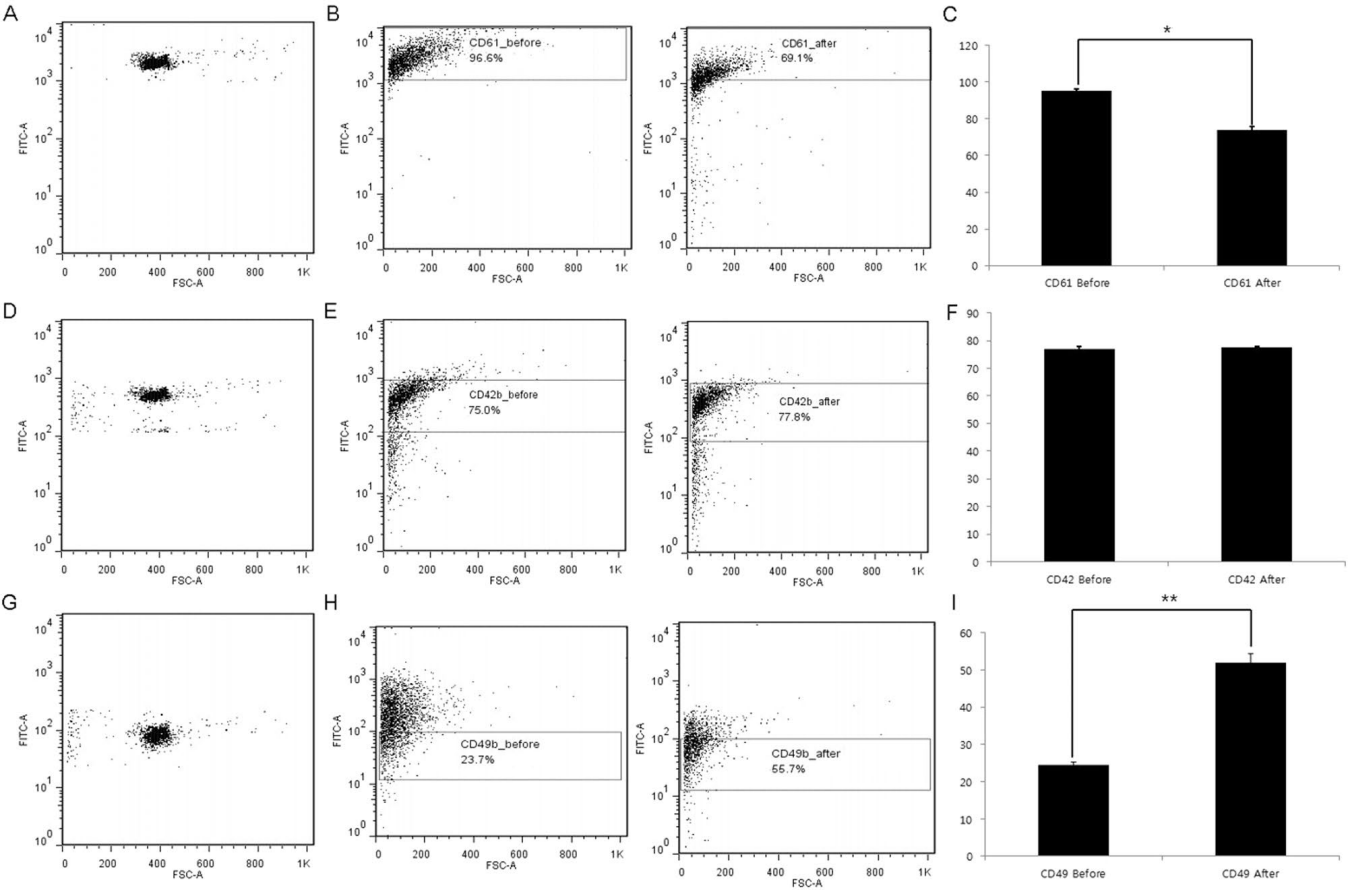
Changes in the expression of platelet surface glycoprotein after *in vitro* exposure to modified charcoal. (**A**) Identification of CD61-positive control platelets. (**B**) Gating of CD61 expression on platelets. CD61 expression was decreased after treatment. (**C**) CD61 content of platelet shown as a percentage of total platelets. (**D**) Identification of CD42b-positive control platelets. (**E**) Gating of CD42b expression on platelets. CD42b expression was not changed after treatment. (**F**) CD42b content in platelets shown as a percentage of total platelets. (**G**) Identification of CD49b-positive control platelets. (**H**) Gating of CD49b expression on platelets. CD49b expression was increased after treatment. (**I**) CD49b content in platelet shown as a percentage of total platelets. *p < 0.05; **p < 0.01.

The proportion of platelets expressing CD61 significantly decreased from 95.2 ± 0.9% to 73.9 ± 1.6 percent (Fig. 4B,C). Platelets expressing CD42b did not show significant changes (77.0 ± 0.9% *vs*. 77.6 ± 0.1%) (Fig. 4E,F). However, the proportion of platelets expressing CD49b increased significantly from 24.6 ± 0.7% to 51.9 ± 2.3 percent (Fig. 4H,I).

### Changes in thrombin-antithrombin complex, fibrin degradation product, and d-dimer

**Figure 5:**
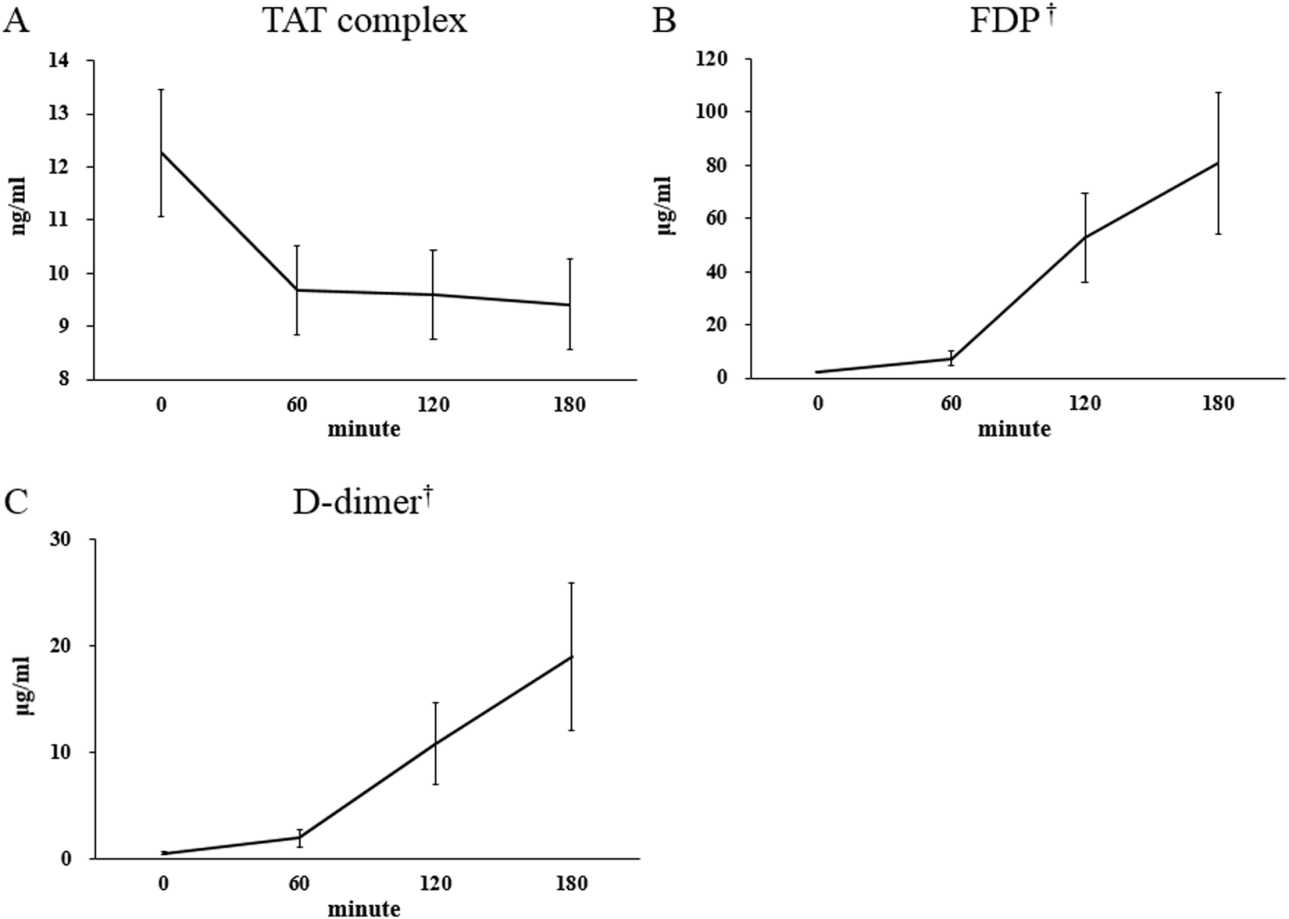
Changes in (**A**) thrombin-antithrombin (TAT) complex, (**B**) fibrin degradation product (FDP), and (**C**) d-dimers during hemoperfusion (*n* = 7, mean ± standard errors). ^†^*p* < 0.05 in Jonckheere-Terpstra trend test.

The results of changes in platelet characteristics observed during HP prompted to perform additional investigations into whether the platelet dysfunction led to impairment of the coagulation process. Thus, we explored the effect of HP on the coagulation process in seven new patients. Although it was not statistically significant, TAT levels, markers of thrombin generation, appeared to decrease during HP from 12.26 ± 1.20 ng/mL to 9.41 ± 0.85 ng/mL (Fig. 5A). Markers of fibrinolysis, including FDP and d-dimers, gradually increased over time during HP with statistical significance (FDP, from 2.18 ± 0.31 μg/mL to 80.72 ± 26.63 μg/mL, Fig. 5B; d-dimer, from 0.50 ± 0.11 to 19.01 ± 6.96 μg/mL, Fig. 5C).

## Discussion

Our data suggest comprehensive mechanistic perspectives of impairment of the hemostatic process occurred during HP. These include reduced platelet counts (Fig. 1), platelet dysfunction (Fig. 2) despite platelet activation (Figs 1 and 3), and increased fibrinolysis (Fig. 5).

After initiation of HP, platelet counts decreased abruptly after 30 minutes, then the rate of decline diminished over time (Fig. 1C). This phenomenon might be because the absorbable area of modified charcoal is the largest at the early period of HP and gradually decreases as HP progresses. Similar changes observed in Hb levels and WBC counts support this hypothesis (Fig. 1B,C). In particular, the abrupt decrease of Hb observed minutes after the initiation of HP (Fig. 1B) was demonstrated by the adherence of red blood cells aggregated with platelets on the surface of modified charcoal in SEM (Fig. 3E).

Interestingly, WBC counts sharply declined in the first 30 minutes then rapidly recovered (Fig. 1A), implying consumed WBCs instantly recovered. Given our results and previous studies associated with hemodialysis and platelet-leukocyte aggregation, it is suggested that similar mechanisms play critical roles in the hemostasis and coagulation occurring during HP^14,15^, and may involve bio-incompatibility^16^. The cause of decreased PCT was attributed to decreased platelet counts, since there were no significant changes in MPV (Fig. 1F). Activation of complement system might also affect leukocyte and platelet-count and the coagulation cascade^17^. Cellulose acetate membranes used in Adsorba 300 C can activate the complement cascade^10^. Unfortunately, we did not measure complement activation in the present study. The initial abrupt decline in WBC counts and more rapid decrease in platelet counts observed in our study could be caused by complement activation in addition to the largest absorbable area at the early period of HP. Although sterilization method could affect biocompatibility, Adsorba 300C is a steam-sterilized device. Steam-sterilized membrane can improve biocompatibility^18,19^.

Our results were different from those from other studies on hemodialysis. In previous studies on hemodialysis using cellulose acetate as a membrane, platelet reduction has been found to be significantly less than that using Cuprophan^R^ membrane. Platelet count not only returns to pre-dialysis level, but also overshoots slightly by the end of dialysis^10^. The hemoconcentration associated with ultrafiltration which occurs during hemodialysis may lead to an increase of platelet count rather than to its decrease. However, HP does not lead to hemoconcentration. Thus, we can explain that platelet reduction in HP does not appear to occur only by complement activation as in hemodialysis. As shown in our study, additional mechanisms might have been involved. Recent studies suggest that extracorporeal cytokine hemoadsorption adds to an optimal level of biocompatibility and its use in HP is not associated with adverse reactions or signs of cytotoxicity^20,21^. The possible impact of roller pumps on platelet count in extracorporeal blood circuits should also be considered. Although it was beyond the aim of the present study, according to a previous study, it can be assumed that roller pumps had no significant effect on our results^22^.

In our study, platelet activation was verified by two methods. First, PDW gradually increased from 41.98 ± 9.28% to 47.69 ± 11.18% during HP (p = 0.021) (Fig. 1D), implying the morphologic changes and platelet activation as reported in previous studies^11^. Second, the activated and adhered platelets on the surface of modified charcoal were observed by SEM (Fig. 3). Despite platelet activation, there was no significant change in MPV (Fig. 1E). MPV is also an indicator of platelet activation and known to increase along with shape changes during platelet activation^23^. Nevertheless, delayed closure time in PFA-100 was observed, suggesting that platelet dysfunction had developed (Fig. 2). These findings suggest that, despite platelet activation and changes in platelet shape which had occurred during HP, the process was not completed and immaturity of platelet storage granules might be associated with platelet dysfunction.

Decreases in Hb and platelet count affect PFA-100 results^13,24^. In the present study, however, there was no significant difference in Hb levels before and after hemoperfusion. In addition, despite platelet counts that decreased significantly after HP, they were higher than 100 × 10^9^/L. Therefore, the change in PFA-100 was considered to actually be platelet dysfunction, rather than an effect of anemia or thrombocytopenia.

Platelets undergo processes of adhesion, activation, and aggregation sequentially in physiologic conditions^25^. Therefore, platelet activation accompanied by reduced platelet aggregation are mutually exclusive processes and suggest that altered platelet glycoprotein expression, not observable in physiologic conditions, had occurred. The results from the *in vitro* study using flow cytometry showed little change in platelets expressing CD42b – vWF receptor (Fig. 4F), and significant reduction in those expressing CD61 – fibrinogen receptor (Fig. 4C). Because the receptors for vWF and fibrinogen play important roles in platelet aggregation, insufficient expression of these receptors supports our hypothesis that an impairment of platelet aggregation had occurred by exposure to modified charcoal. In contrast, expression of CD49b, collagen receptor, increased (Fig. 4I), suggesting that platelet activation would be stimulated by the signaling pathways induced by platelet adhesion. In the context of rheology, the degree of shear rate in the HP circuit was not evaluated. However, it could be supposed that the blood passing through the HP circuit lies on a low shear rate. During HP, blood does not pass through a hollow fiber with a narrow caliber as it does in HD, and the blood flow rate is lower than in HD. Thus, it has been suggested that platelet adhesion is closely related to the expression of collagen receptors on the surface of platelets^26^.

Currently discussed coagulation pathways based on a concept of a cell-based model. It has replaced the classical “waterfall” cascade model^27–29^. In this model, platelets are needed for the initiation of thrombin generation. Platelet activation leads to activation of platelet integrin α_IIB_β (GPIIb-IIIa). Because inhibition of GPIIb-IIIa by “abciximab” antibodies or by the glycoprotein IIb/IIIa inhibitor “eptifibatide” leads to decreased thrombin generation^30,31^, changes shown in our study might affect the coagulation process, including thrombin generation^32^. Thus, levels of TAT, markers of thrombin generation, and FDP and d-dimers, those for fibrinolysis, were measured. The level of TAT appeared to decrease gradually as HP progressed, but this change was not statistically significant (Fig. 5A). In contrast, levels of FDP and d-dimers increased significantly over time (Fig. 5B,C). These results suggest increased fibrinolysis occurred concomitant with decreased thrombin generation^33,34^. The changes in the coagulation process were considered to result in bleeding complications combined with platelet aggregation dysfunction. Taken together, our data suggest that hemoperfusion led to impairment of platelet aggregation with incomplete platelet activation, which was associated with reduced thrombin generation accompanied with increased fibrinolysis.

Our study had several limitations. First, because participants ingested pesticides and suffered from various toxic symptoms, effects of the pesticides on the process of hemostasis could not be excluded. Second, at the beginning of the study we actually focused only on platelet dysfunction which occurred after HP. However, results of the studies on platelet function prompted an additional evaluation of the coagulation process. Thus, we evaluated the coagulation process with an additional seven patients who were not involved in the study for platelet function because it might be ethically problematic to draw too much blood from the patients who underwent HP. Therefore, this part of the study was not experimentally sequential and the results may not be relevant, because it was based on samples from different patients. Third, all patients received heparin for anticoagulation during HP. The inevitable use of anticoagulation may have affected our results. Fourth, the present study examined impairment of the hemostatic process that occurred during HP. Therefore, our results only showed an overall view of impairment of the hemostatic process, rather than causality, and mechanistic conclusions are ambiguous. Finally, we did not check for the activation of the complement system in our investigations, although complement activation may have an impact on our results.

In conclusion, our data showed that hemoperfusion led to impairment of platelet aggregation with incomplete platelet activation, which was associated with reduced thrombin generation accompanied with increased fibrinolysis. Our results could provide direction for further studies on bleeding complications that occur from HP.
